# Batch and Flow Ultrasound-Assisted Extraction of Grape Stalks: Process Intensification Design up to a Multi-Kilo Scale

**DOI:** 10.3390/antiox9080730

**Published:** 2020-08-10

**Authors:** Giorgio Grillo, Luisa Boffa, Salvatore Talarico, Roberto Solarino, Arianna Binello, Giuliano Cavaglià, Samir Bensaid, Galina Telysheva, Giancarlo Cravotto

**Affiliations:** 1 Dipartimento di Scienza e Tecnologia del Farmaco, University of Turin, Via P. Giuria 9, 10235 Turin, Italy; giorgio.grillo@unito.it (G.G.); luisa.boffa@unito.it (L.B.); s232314@studenti.polito.it (S.T.); roberto.solarino@unito.it (R.S.); arianna.binello@unito.it (A.B.); 2 DISAT—Polytechnic of Turin, C.so Duca degli Abruzzi 24, 10129 Turin, Italy; gcavagli@libero.it (G.C.); d019403@polito.it (S.B.); 3 Latvian State Institute of Wood Chemistry, 1006 Riga, Latvia; ligno@edi.lv

**Keywords:** ultrasound-assisted extraction, grape stalks, polyphenols, process intensification, extraction kinetics, kilo-scale procedure, flow system, antioxidant activity

## Abstract

Nowadays, approximately 1 billion kg/y of grape stalks, with a remarkable polyphenols content, are produced worldwide. In this paper, the extraction process intensification of polyphenols in water was achieved under ultrasound-assisted recovery, focusing on kinetics and scaling-up factors. Immersion and cup-horn systems were exploited as acoustic cavitation sources, and the total phenolic content (TPC) was chosen to assess the process efficiency. The kinetics were evaluated by Peleg’s hyperbolic model, and the effect of both the initial feedstock granulometry and ultrasound size-reduction were determined. The results were compared with conventional extraction methods (data analysis by ANOVA). The best polyphenols yield was obtained after 45 min of sonication, giving between 29.71 and 31.89 mg/g (gallic acid equivalents over the dry matter). The extracts were characterized using HPLC-DAD, UPLC-ESI-MS/MS, DPPH^•^ assay (2,2-diphenyl-1-picrylhydrazyl), TEAC assay (Trolox equivalent antioxidant capacity), and proanthocyanidin content determination. The flow-mode extraction procedure of grape stalks (2 kg) was carried out in a 15 L reactor. A semi-industrial decanter unit and a bag-filter were the keys units of the downstream operations. The resulting particle-free solution underwent nanofiltration on a membrane pilot skid, providing a final polyphenols-enriched stream concentrated up to 355.91%, as shown by the antioxidant activity and TPC.

## 1. Introduction

Viticulture (*Vitis vinifera* L.) is one of the world’s most important agricultural activities, producing up to 77 million tons of grapes every year. Around 75–80% of this crop is used to produce wine, which, with a demand of 25 billion liters per year, has earned the title of the most important alcoholic beverage in the world [1]. The production process, mainly located in Europe and America, followed by Australia, China, and South Africa [2], starts from the seasonal grape harvest and then proceeds to operations that produce the finished product, which can be diversified according to the various characteristics requested. However, winemaking also involves the parallel production of large quantities of processing waste, from the destemming, pressing, settling, and washing operations, and thus has a significant environmental impact and disposal costs. On the one hand, it has been estimated that for each liter of wine produced, one to several liters of wastewater are derived. On the other hand, the amount of solid residue resulting from this supply chain, which includes grape marc and stalks, is equally impressive, and reaches 5 tons per year for each hectare cultivated [3,4]. Stalks make up between 2.5% and 7.5% of the grape weight, depending on the grape cultivar and pressing methods, and about 13% and 16% of white- and red-wine solid waste, respectively. Besides being essentially composed of cellulose and lignin [5,6], the grape-bunch skeleton also contains lipophilic compounds (e.g., sterols and triterpenes) [7] and polyphenols, which can be divided into flavonoids, e.g., flavonols, anthocyanins, and flavan-3-ols (monomer, dimers, oligomers, and polymers), and non-flavonoids, mainly phenolic acids and stilbenes [8,9]. All of these compounds can have a beneficial influence on human health, including anti-carcinogenic, anti-atherogenic, and anti-mutagenic effects; protection against cardiovascular diseases; and anti-inflammatory activity [10]. Moreover, it has been shown that winery by-products can be a good source of natural preservatives, because they contain molecules with antibacterial and antioxidant properties, such as hydroxycinnamic acid derivatives, tannins, catechins, and the other above-mentioned phenols [11]. The shelf-life of food products, especially when they contain oils and fats, can therefore be prolonged by the addition of natural and safe secondary metabolites of wine [12].

Although the stalk fingerprints for the polyphenols composition and concentration depend on several factors, including grape variety, climate, time of harvest, and geographic origin, there is important commercial value in this type of purified bioactive molecule [13]. In fact, we are currently witnessing a constant increase in the number of drugs, cosmetics, and nutraceutical-product formulations that are making a high antioxidant power, and therefore high polyphenolic content, one of their main strengths. The exploitation of this common waste as a negative cost feedstock may provide a significant economic advantage. Industrial waste valorization is now, therefore, a mandatory objective as a means to decrease pollution and simultaneously recover compounds with a high added-value, such as polyphenols, which would otherwise be lost, often with detrimental effects on the flora and fauna in the affected areas.

Agro-food industry waste treatment often involves the extraction and purification of natural compounds that can be employed in functional foods, nutraceuticals, bio-fuels, cosmetics, drugs, and other bio-products [14,15]. The management of winery and grape-juice residues is related to the high content of organic matter as well as the large amount generated over a short period of the year, with the consequent environmental problems of biological and chemical oxygen demand [16]. The “green chemistry” concept is strictly connected to the prospect of a cleaner and more sustainable world, and includes any practice that aims to reduce the use of hazardous and polluting substances and any waste released into the environment, and thus supports any action that enables the reuse, treatment, and disposal of such materials. Synergistically, “green engineering” bases its principles on ensuring the design of plants with reduced risks, taking into account not only the reliability of the control systems, but also ensuring maximum safety. The 12 principles of green chemistry and the 12 principles of green engineering, which can be summarized by the acronyms “IMPROVEMENTS” and “PRODUCTIVELY”, respectively, can be combined in order to allow for improvements in production together with reliable scale-up criteria [17].

The idea of optimizing extraction techniques, also when used to valorize agro-food wastes, fits perfectly with these concepts. It is important to revolutionize conventional procedures with the purpose of intensifying processes, while also enhancing their sustainability. Chemat et al. [18] have indicated what needs to be done in designing a competitive extraction process, namely: innovation, flexibility, the use of alternative solvents (mainly water), reduction in energy consumption, co-product production instead of waste, process safety, limited operative units, and the avoidance of contaminants. Extreme attention must also be paid to the downstream separation and purification steps. Improving existing processes involves the use of enabling technologies, which can provide the two-fold effects of increasing efficiency, while greatly reducing the environmental and economic impact [19]. As shown in the high number of recently published works (about 5000 only in the last 5 years), ultrasound-assisted extraction (UAE) is now fully recognized as a green technique by the pharmaceutical, cosmetic, and food industries [20]. Only physical effects are exerted on an immersed/dispersed matrix when ultrasound (US) waves cross a medium at a frequency of 20–100 kHz, because they only exert a mechanical action at a low frequency and high intensity. In addition to the sonotrode used in the extraction processes, either immersion and cup-horn systems, a transducer is used to transform the signal of a generator into mechanical waves, with compression/rarefaction cycles in the US frequencies, giving rise to the well-known cavitation phenomenon. Several physical phenomena develop as a consequence of this effect, and macro-turbulence and micro-mixing are generated in the case of a liquid–solid system. The synergy of several independent factors, including fragmentation, erosion, capillarity, destructuring, and sonoporation, lead to the disruption of vegetal matrices and to a higher solvent-penetration coefficient, thus significantly improving mass transfer kinetics during an extraction procedure [20,21]. Operating times are reduced, the purity of the final product is increased, and, furthermore, the plant can be simplified by reducing the (L/S) ratio, leading to reductions in the solvent amount and the minimization of wastewater post treatment. It is worth noting that all of these goals can also be achieved when working at room temperature and using a fraction of the energy required for a conventional extraction process, which, in the case of solid waste from wine making, commonly involves alcohol or hydro alcoholic mixtures and heating above 90 °C [22].

The grape stalks are essentially recycled as fertilizer [23], and the possibility of their valorisation to produce health-promoting compounds on an industrial scale must still be investigated.

The present work has screened polyphenols UAE from grape stalks in water and has focused on extraction kinetics and scale-up factors. The total phenolic content (TPC) was chosen as the parameter to rapidly assess process efficiency. The results have been compared with those of the conventional extraction and of runs with different feedstock-size distributions. Peleg’s hyperbolic model was used to describe the UAE kinetic curves in lab-scale apparatus, and, consequently, to determine the best trade-off between the extraction rate and process time. The related volumetric mass transfer coefficient (kLa) was crucial for the investigation of the process scale-up design, up to an industrial-plant scale, with a multistage UAE section. A flow approach, in a 15 L reactor and using a 120 L tank in recirculating extraction, processed between 2 to 6 kg of grape stalks. The scaled-up protocol required dedicated facilities for the post-extraction treatment, such as a decanter unit and a bag-filter (150 µm mesh), and the resulting particle-free solution underwent treatment on a nanofiltration (NF) membrane pilot skid in order to give a concentrated polyphenols-rich fraction. Lab-scale extracts were characterized using HPLC-DAD, UPLC-ESI-MS/MS, DPPH^•^ scavenging activity (2,2-diphenyl-1-picrylhydrazyl assay), TEAC assay (Trolox equivalent antioxidant capacity), and proanthocyanidin content determination. The antioxidant activity and TPC were used to follow the efficiency of the UAE scale-up from the lab- to pilot-scale.

## 2. Materials and Methods

### 2.1. Chemicals

EtOH (ACS grade, ≥99%), methanol (MeOH, HPLC, ≥99.9%), and acetone (ACS grade, ≥99%), used for the extractions and micro-assays, and acetonitrile, used for the HPLC analyses (MeCN, HPLC Plus, ≥99.9%), were purchased from Sigma-Aldrich (Sigma-Aldrich, Milan, Italy), while Milli-Q H_2_O was obtained in the laboratory using a Milli-Q Reference A + System (Merck Millipore, Darmstadt, Germany). Glacial acetic acid (AcOH, ≥96%) was purchased from Merck (Darmstadt, Germany).

Analytical standards of gallic; gentisic; *o*-hydroxybenzoic (salicylic); *p*-hydroxybenzoic; protocatechuic; vanillic; 3,4-dimethoxybenzoic (veratric); syringic, ellagic; cinnamic; *o*-, *m*-, and *p*-coumaric; caffeic; chlorogenic; ferulic; 3-hydroxy-4-methoxycinnamic; 3,4-dimethoxycinnamic; 3-methoxycinnamic; 4-methoxycinnamic; and sinapic acids, as well as (+)-ε-viniferin, (−)-epicatechin, quercetin-3-glucoside, apigenin-3-glucoside, and luteolin-3-glucoside for the HPLC analyses were purchased from Sigma-Aldrich. The Green Tea Catechin Mix (100 μg/mL caffeine, (+)-catechin, (−)-catechin 3-gallate, (−)-epicatechin, (−)-epicatechin-3-gallate, (−)-epigallocatechin 3-gallate, (−)-gallocatechin, and (−)-gallocatechin 3-gallate in acetonitrile:water (8:2) with 5% 1M HCl, Cerilliant^®^, Sigma Aldrich) was used for the HPLC analyses of catechins.

A procyanidin B2 standard, sodium metabisulfite, *n*-butanol, and hydrochloric acid for the proanthocyanidin content determination; Trolox^®^ and the 2,2-diphenyl-1-picrylhydrazyl radical (DPPH^•^) for antioxidant activity determination; and the Folin–Ciocalteu reagent, sodium carbonate, and DMSO for the total polyphenol content (TPC) determination were all purchased from Sigma Aldrich.

### 2.2. Matrix Analysis

Dried and milled grape stalks were kindly provided by Cantine Ascheri Giacomo (Bra-CN, Italy), obtained by mechanical separation from grapes. The resulting material was not submitted to any dedicated drying process. The matrix was stored at room temperature until the extraction procedures.

The water content and the organic and ashes fractions were obtained by means of thermogravimetric analysis. First, 200 mg of raw grape stalks were maintained at 100 °C over night, in order to register the biomass water content as a mass loss, and then for 4 h at 650 °C so as to allow for discriminating between the organic and inorganic residual content.

The matrix granulometry was analyzed by means of physical separation. An average quantity of 7.5 g of grape stalks was passed through sieves with decreasing mesh sizes (Giuliani, Italy 1000, 500 and 212 µm). The different weights were registered and applied for percentage compositions.

### 2.3. Lab-Scale Ultrasound-Assisted Extraction (l-UAE)

l-UAE was performed using two different US devices, an immersion horn (I-horn, HNG-20500-SP, Hainertec Suzhou, China) and a cup-horn (C-horn, PEX1, R.E.U.S., France). The two systems work under different conditions, namely: 500W of power at a frequency of 21 kHz and 200 W of power at a frequency of 25 kHz, respectively.

In the function of the extraction vessel volume, the experiments were performed by mixing 7.5 g of grape stalks (<1000 µm and 212 µm) with the correct amount of deionised water in order to achieve an S/L ratio of 1:20. For the I-horn, the solution was placed in a Pyrex^®^ thimble (r: 4 cm and h: 19 cm), while the C-horn did not require other glassware. The temperature was registered throughout the UAE, and heat exchange was provided using two different approaches—an ice bath for the I-horn and a cooling jacket with a continuous flow of tap water for the C-horn. Where required, an extraction temperature of 45 °C was achieved via US-derived heating for a few minutes. For this purpose, the cooling system was removed until the fixed limit was reached.

After extraction, the solutions were vacuum filtered, and the matrices were thoroughly washed with deionized water. The crude extracts were then freeze dried (LyoQuest–85, Telstar, Spain), and the dry material was weighted and analysed for the total polyphenol content (TPC) determination. The optimized extract (C-horn, 45 °C, 45 min) was also characterized using HPLC analyses, antioxidant activity, and oligomeric proanthocyanidin (OPC) content assays.

### 2.4. Conventional Extractions

For the sake of comparison, three types of conventional approaches were also carried out, namely: (1) silent extraction, (2) water reflux multi-step extraction, and (3) a reflux hydroalcoholic benchmark. For (1), the optimized parameters for l-UAE were exactly reproduced without US irradiation. In approach (2), 7.5 g of stalks were mixed with 150 mL of deionised water (S/L 1:20), and were extracted for 180 min at a reflux temperature. The matrix was then recovered, washed, and extracted twice under the same conditions. The crude extracts derived from procedures (1) and (2) were freeze dried and analysed separately for the TPC. Finally, extraction (3) was conducted by reproducing the hydroalcoholic extraction as found in the literature [22] for <1000 µm and 212 µm granulometries. Then, 7.5 g of the selected matrix was mixed with 150 mL of a hydroalcoholic solution (S/L ratio of 1:20, EtOH/H_2_O 60:40), and heated for 23 min at 95 °C under stirring. The matrix was then filtered and washed three times with a fresh extraction solvent. The crude extracts underwent the sequential removal of EtOH and water by means of rotary evaporation and lyophilisation, respectively. The dry product was analyzed for TPC determination, and for the reference sample (hydroalcoholic mixture), HPLC analyses, antioxidant activity, and OPC assays were performed.

### 2.5. Scale-Up Ultrasound-Assisted Extraction (S-UAE)

S-UAE was performed using a Biopush flow-through cell reactor from Weber Ultrasonics GA (Germany), which is able to simultaneously process 15 L of solution under irradiation at up to 2000 W, at a frequency of 29 kHz. In order to reflect the information obtained under the l-UAE optimized conditions as closely as possible, the system was used in a loop configuration. The loop also included a squared section (460 × 460 mm) with a 45° pyramidal bottom 120 L tank, equipped with an impeller with four inclined blades (blade inclination: 45, impeller diameter: 200 mm, impeller position: 110 mm above pyramidal cone starting plane, installed power: 0.18 kW, rotational speed: 1340 rpm) to form the solid–liquid suspension and to maintain it in a homogeneous state, so that it could be pumped by the dedicated progressing cavity pump (MAE 50-1/AA. T33A, CSF, Italy).

**Figure 1:**
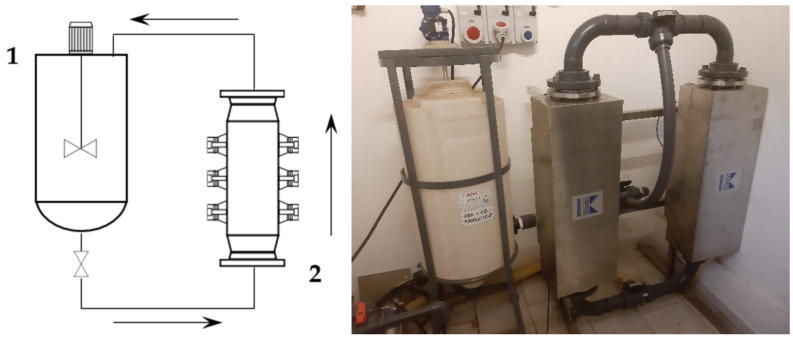
Recirculating flow system. Left: set-up scheme (1: mixed tank, 2: ultrasound (US) flow-through cell); right: facility picture.

First, 2 kg of sieved grape stalks (<1000 µm) were mixed with water at 45 °C to reach the desired S/L ratio of 1:30, for a total addition of 60 L. The pump speed was set to 300 rpm, resulting in a flow rate of 30 L/min. The recirculated solution entered the middle section of the tank, and thus provided additional suspension mixing together with the blade mixer. The tank acted as a 45 L buffer while 15 L were processed. The ultrasonic reactor BioPush Weber, with a generator input power of 2000 W, was used for all of the S-UAE, thus allowing for an automatic time control to be set. Then, 50–100 mL of samples were collected at fixed times. A schematic representation and relative picture can be found in Figure 1.

At the end of the sonication, the suspension was pumped into a dedicated 250 L tank and the extraction system was thoroughly washed. The resulting solution was separated by means of an F2000 decanter (Andritz, The Netherlands), running at a differential speed of 7 rpm and a pipette position of 10 cm. The system was fed with a 500 kg/h flow, and was finally washed with water to complete the extract recovery.

The solid stalk residues were collected below the decanter, while the liquid fraction was recovered in a separate tank and sent to concentration through NF. The samples were collected and freeze dried according to the l-UAE protocol. All of the collected samples were dried and then analyzed for TPC determination.

### 2.6. Nanofiltration Concentration (NF Concentration)

The crude extract recovered from S-UAE amounted to approximately 100 L, and was processed by NF performed on a HARI P2B200-400 membrane skid (HydroAir Research, Lodi, Italy) that was equipped with a DLU 19 F cartridge (2.5 m^2^) with an approximative MWCO (molecular weight cut-off) span of 150–300 Da. The flow rate was set to 1000 L/h by means of a by-pass and counter-pressure regulation. The latter was raised to 10 bar and maintained until a constant output was observed. Once the residual volume reached 40 L, the pressure was increased to 15 bar for the remaining time. The temperature was monitored, and ranged from 25 to 30 °C. The process was concluded when precipitation due to solvent removal was incipient, at a residual volume of roughly 20 L. The concentrated fraction corresponded to the retentate stream, continuously recycled into the feed tank, while the removed aqueous solution converged into the permeate output. The retentate was collected during the process and the dry extract (DE) yield was derived by freeze drying. The NF trend was defined by the percentage global concentration (GConc) and instant concentration (InstConc). GConc considered *t* = 0 dry yield as a reference, while InstConc provided a ratio between two adjacent samples. An aliquot of the permeate was also freeze dried so as to determine the mass balance.

### 2.7. Total Phenolic Content (TPC)

TPC was determined according to the method developed by Cicco et al. [24]. Quantification was carried out using a standard curve of gallic acid as the reference phenolic compound, at dilutions of between 5 and 250 µg/mL in a H_2_O/DMSO 1:1 mixture. Dried samples were dissolved in a H_2_O/DMSO 1:1 mixture at a concentration of nearly 0.5 mg/mL. The gallic acid and extract solutions (250 µL) were placed into test tubes. The following solutions were added sequentially to each tube: 250 µL of Folin–Ciocalteu (diluted 1:1 with distilled H_2_O), 500 µL of a 10% *p/v* Na_2_CO_3_ solution, and 4 mL of distilled H_2_O. The resulting solution was vigorously shaken and left at room temperature for 25 min prior to the analysis. The absorption of the final mixtures was measured at 740 nm, in a 1 cm cuvette using a Cary 60 UV-VIS spectrophotometer (Agilent Technologies, Santa Clara, CA, USA). These conditions provided the assay with a high accuracy and reproducibility. The TPC was expressed as gallic acid equivalents (GAE; mg/g) over the dried matrix (DM). All of the analyses were performed in triplicate and were expressed as averages. The standard errors (SE) are specified in the tables at confidence level of 0.95.

### 2.8. Peleg Method

Peleg’s hyperbolic model (see Equation (1)) [25] was applied to evaluate the extraction kinetics and to determine the point of maximum extraction rate by means of the related constants.

(1) $$C\left(t\right)=C_{0}+\frac{t}{k_{1}+k_{2}t}$$

C(t) is the concentration of the extract after extraction time t, while C_0_ is equal to 0 at the beginning of the process. The Peleg initial extraction rate (k_1_) is correlated to the starting extraction rate (B_0_, Equation (2)), and can be used to calculate the relative extraction rate at each moment of the extraction (B_t_).

(2) $$B_{0}=\frac{1}{k_{1}}$$

B_o_ allows the maximum extraction rate achievable when the mass-transfer driving force is at its maximum to be calculated (i.e., when the difference in the solute concentration in the solid matrix and in the liquid extractant is at its maximum), which is an important factor for cross-flow and counter-current industrial-scale extraction processes.

The Peleg capacity constant (k_2_) is correlated to the highest extraction yield achievable at the steady state with a single equilibrium stage (Y_s_, Equation (3)). It is also important data that are needed to define the number of extraction stages that the industrial process must have in order to achieve the required overall extraction yield. From a graphic point of view, this parameter represents a horizontal asymptote.

(3) $$c_{0}=c_{eq}=Y_{s}=\frac{1}{k_{2}}$$

**Figure A1:**
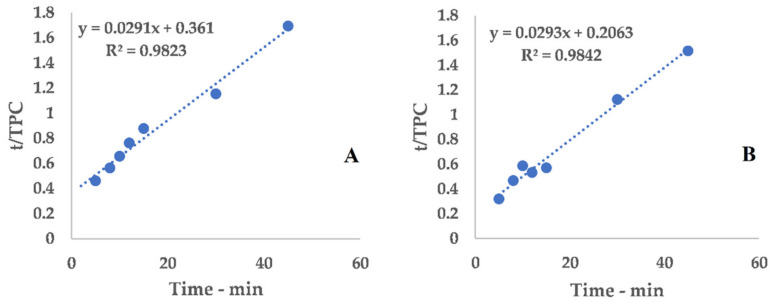
Linearization, I-horn; (**A**): RT; (**B**): 45 °C.

**Figure A2:**
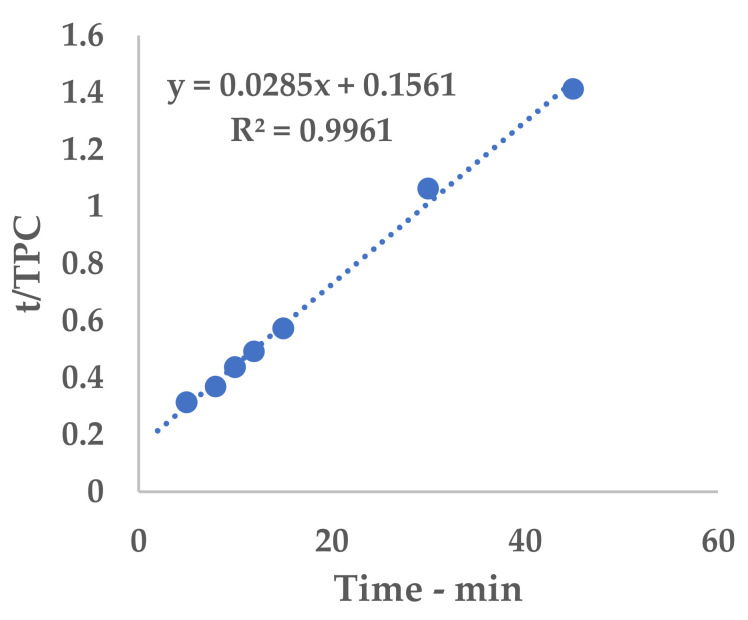
Linearization, C-horn.

Equation (1) can be conveniently linearized into Equation (4), thus providing a fast and easy way to extrapolate k_1_ and k_2_ as the intercept and slope, respectively. The kinetic constants can be calculated via the linear interpolation of the experimental yields at different extraction times (see Table A1 and Figure A1 for 6 mm, and Table A2 and Figure A2 for 2 mm), and then filled into Equation (1).

(4) $$\frac{t}{\;C\left(t\right)}=k_{1}+k_{2}t$$

The obtained hyperbolic curve describes a time-dependent extraction trend. This model is useful to display the horizontal asymptote of Y_s_ and the extraction rates (slope of the curve). Furthermore, the so-called “knee-point” can be exploited to determine the best trade-off between productivity and the process extent. This graphical feature can be identified where the extraction efficiency starts to steeply increase and it no longer worth the time consumption. In the present work, the abovementioned parameter could not be directly applied because of the necessary technological transposition (see Section 3.5).

### 2.9. Antioxidant Activity (DPPH^•^ Radical Scavenging Method and TEAC)

The radical scavenging ability of the extracts was evaluated using the stable free radical DPPH^•^, according to the method described by Brand-Williams, Cuvelier, and Berset [26]. Details of the procedure and calculations have been reported by Boffa et al. [27]. The bleaching rate of the DPPH^•^ radical was monitored in the presence of solutions of grape-stalks extracts, as well as in that of Trolox^®^ (antioxidant standard for TEAC determination), in order to calculate the EC_50_ (half maximal effective concentration or amount of compound/extract necessary to decrease the initial concentration of DPPH^•^ to 50% at equilibrium). The radical scavenging activity of the Trolox^®^ solutions was measured in concentrations from 1 to 18 µg/mL, corresponding to 0.004, 0.008, 0.016, 0.020, 0.028, 0.040, 0.048, and 0.072 µmol/mL. Various concentrations of grape-stalk extracts, between 5 and 500 µg/mL, were also tested. All of the samples were prepared in triplicate, and the DPPH^•^ radical scavenging activity was expressed as µg compound/dried extract per mL solution ± standard deviation. The Trolox^®^ equivalents (TE) µmol/g of the extract were calculated according to the EC_50_ values.

### 2.10. Oligomeric Proanthocyanidin Content (OPC)

The OPC in the extracts obtained using l-UAE (C-horn, 45 °C, 45 min) and conventional hydroalcoholic extraction (23 min, 95 °C under stirring) was quantified using the butanol-HCl method with procyanidin B2 as a reference [28]. Briefly, the procedure by Schofield et al. includes the serial dilution of samples (or standard), followed by the addition of an acid butanol reagent (*n*-butanol/concentrated HCl, 95:5 by volume) and iron reagent (2% ferric ammonium sulphate dodecahydrate salt, FeNH_4_(SO_4_)_2_·12H_2_O in HCl 2 M). After 50 min at 95 °C, the absorbance was registered at 550 nm on a Cary 60 UV-VIS spectrophotometer (Agilent Technologies, Santa Clara, CA, USA).

### 2.11. HPLC-DAD Analysis

The HPLC analyses were performed on a Waters 1525 pump linked to a 2998 PDA (Waters Corp., Milford, CT, USA), using a Synergi Hydro RP C18 column (250 mm, 4.6 mm, 4 μm; Phenomenex, Torrance, CA, USA), with 2% AcOH (A) and MeCN (B) as the mobile phases. The monitored wavelengths were 280 (benzoic acids, catechins, and proanthocyanidins detection) and 340 nm (cinnamic derivatives and flavonoids detection), while three-dimensional data were acquired in the 200–600 nm range. Two different methods were used. The first gradient program (Pol) started from 0% B, which was maintained for 6.5 min, up to 50% B over the 6.5–30 min period, from 50% to 100% B for 30–36 min, followed by a 100% B step at 36–42 min. The second method (Cat) started from 8% B, which was maintained for 3 min, up to 50% B over the 3–21 min period, from 50% to 100% B for 21–33 min, followed by a 100% B step at 33–45 min.

Several compounds were screened, as described in Section 2.1. The quantification of polyphenols was performed using calibration curves obtained with external standards. The analytical standard solutions were analysed using HPLC (20 μL injection) to give linear regressions with *R*^2^ > 0.999. The equation curves, the wavelength used for the quantification, R^2^, linearity range, limit of detection (LOD), and limit of quantification (LOQ) for each standard analysed are indicated in Appendix A. The extract samples (l-UAE, C-horn, and conventional hydroalcoholic extraction) were dissolved in H_2_O/MeOH 8:2 before injection (20 μL), giving concentrations of 5–10 mg/mL.

### 2.12. UPLC-ESI-MS/MS Analysis of Polyphenols

An Acquity UPLC system (Waters Corp., Singapore), coupled with a quadrupole-time of flight (Q-TOF) MS instrument (UPLC/Synapt Q-TOF MS, Waters, Milford, MA, USA) and an electrospray ionization (ESI) source, was used. The UPLC column (2.1 mm × 50 mm i.d., 1.7 µm, BEHC18, Waters Acquity) was used at a flow rate of 0.35 mL∙min^−1^. The mobile phases were water with 0.1% formic acid (A) and acetonitrile (B). The gradient program was 0–1 min, 5–15% (B); 1–5 min, 20–25% (B); 5–6 min, 25–75% (B); and 6–7 min, 75–80%. The injection volume was 1 μL. The major operating parameters for Q-TOF MS were set as follows: capillary voltage, 2.5 kV; cone voltage, 60 V; cone gas flow, 100 L/h; source temperature, 120 °C; desolvation temperature, 400 °C; collision gas, argon; desolvation gas, nitrogen; flow rate, 600 L/h; data-acquisition range, *m/z* 50–1200 Da; and ionization mode, negative. The detected compounds were identified by means of MS fragmentations, according to the data reported from a previous work [29].

### 2.13. Statistical Analysis

The statistical analyses were performed using R software (R Foundation for Statistical Computing, Vienna, Austria), version 3.6.3 (2020-02-29). The analyses were all performed in triplicate. Residuals were checked for normality using the Shapiro–Wilk and Kolmogorov–Smirnov tests, and variances were checked for homogeneity using Levene’s test.

The data in Section 3.2.3 and Section 3.3.1 were processed by ANOVA statistical techniques. In details. Single way, two-way (two factors, 3 × 2 levels), and three-way (three factors, 3 × 2 × 2 levels) were investigated to find significant interactions (*p* < 0.01). Tukey’s HSD post-hoc multiple comparison test was used with the Bonferroni adjustment, a confidence level of 0.95, and a significance level α = 0.05 in order to determine the differences between the different sample means. Multiple comparisons were made one factor at a time. The confidence level and *p* value adjustments with the Bonferroni method were based on the number of tests and estimates performed (two or three, based on the factor considered).

A linear model (two-way ANOVA, two factors, 3 × 2 levels) was applied in order to determine the contribution of the time and granulometry, and their interaction (*p* < 0.001). Different linear regressions for the extracted TPC were calculated according to the different granulometries in the time. The SE are specified in the tables at a confidence level of 0.95.

## 3. Results

### 3.1. Grape-Stalk Characterisation

The grape stalks were characterized for their water content, organic/inorganic composition, and size distribution (Table 1). The thermogravimetric analysis revealed that the biomass was efficiently desiccated, with a residual humidity of 8.92%. This value was used to normalize the yields of the following tests, with the TPC and dry extracts being expressed in relation to the dry matrix (DM). The inorganic residues represented 4.46% of the overall stalk quantity, with the organic fraction, to which secondary metabolites and extractables belong, therefore resulting in 86.63%.

Physical separation by means of sieves (1000, 500, and 212 µm) was then used to roughly determine the particle dimensions (Table 1). The main fraction (46.7%) included >1000 µm components and was characterized by a filamentous aspect, forming wad-like agglomerates. Smaller particles, with more rigid behavior, were primarily made up of the 1000 µm (40.0%) granulometry, followed by minor quantities of 500 and 212 µm granulometries.

### 3.2. Ultrasound-Assisted Extraction: Lab-Scale (l-UAE)

The UAE technique was explored on the lab-scale level on two different devices, namely an I-horn and a C-horn. The TPC evaluation was registered over a 45 min time span in order to extrapolate a kinetic model. Tests were performed on particle sizes smaller than 1000 µm, so as to avoid agglomeration and matrix segregation. The screenings reported in Section 3.2.1 and Section 3.2.2 were therefore focused on < 1000 µm granulometries, while Section 3.2.3. reports a comparison with smaller particles (212 µm).

#### 3.2.1. Immersion Horn (I-horn)

The I-horn’s structure means that it requires an external cooling system in order to control the temperature increase that is caused by the cavitation phenomena. Modifying the ice bath temperature allowed the process to be operated at room temperature (RT) or, alternatively, enabled the heat generated by the system to be exploited to reach and maintain an average temperature of 45 °C. The latter condition was tested to verify the synergy between US irradiation and temperature. For each set of tests (RT: 25 °C and 45 °C), the TPC and DE yields at different extraction times are reported in Table 2. A rough idea of extraction selectivity can be obtained from a comparison between the abovementioned parameters.

**Figure 2:**
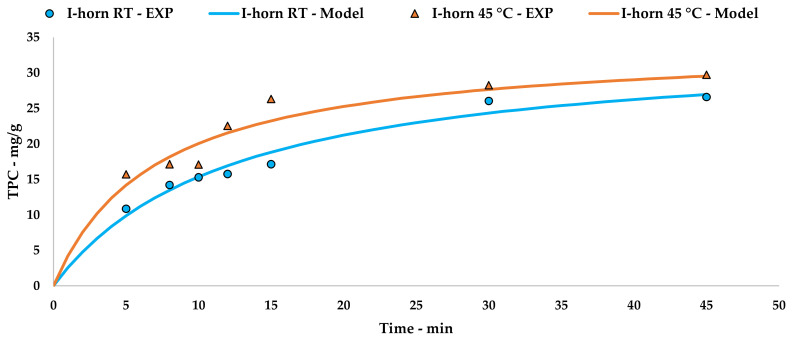
I-horn UAE at different temperatures: experimental values and the Peleg model.

The collected data were processed using Peleg’s method via an extrapolation of the kinetic curve to describe UAE trends (Figure 2, Equations (5) and (6)). The linearization and related tables are reported in Appendix A (Table A1 and Figure A1).

(I-horn at RT)

(5) $$C\left(t\right)=\frac{t}{0.3610+0.0291\;t}$$

(I-horn at 45 °C)

(6) $$C\left(t\right)=\frac{t}{0.2063+0.0293\;t}$$

#### 3.2.2. Cup-Horn (C-horn)

As the TPC trends in Section 3.2.1. defined the protocol at 45 °C to be the best performing I-horn extraction, this temperature was considered for the kinetic screening of the C-horn, and the results are reported in Table 2.

**Figure 3:**
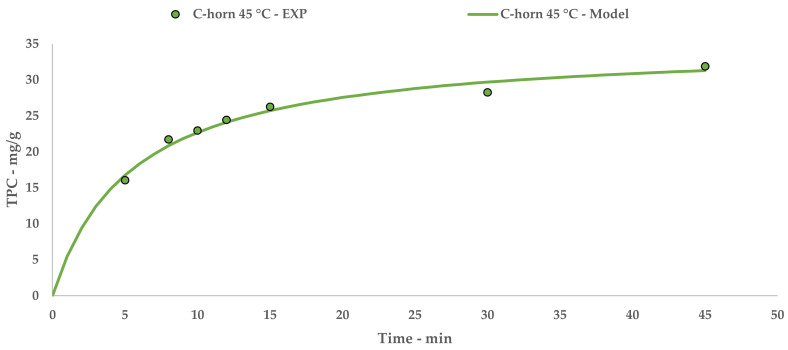
C-horn UAE: experimental values and Peleg model.

The Peleg theoretical kinetic curve is reported in Figure 3, while Equation (7) contains the relative constants. The linearization and related table are reported in Appendix A (Table A2 and Figure A2).

(7) $$C\left(t\right)=\frac{t}{0.1561+0.0285\;t}$$

#### 3.2.3. Granulometry Effect

Both the cup- and immersion-horn were tested for 212 µm fraction extraction using the previously optimized conditions, and the influence of the particle size on UAE was thus evaluated. For the sake of comparison, the I-horn was operated at both RT and 45 °C. The results, and variations compared to <1000 µm particles, are reported in Table 3.

The results suggest that an appreciable size-reduction effect, influenced by the sonotrodes used, occurs at RT. I-horn gave the best performance. To further deepen this aspect, a time-dependent trend was explored for the I-horn at RT, in which the effect appeared to be maximized. The results are reported in Table 4 for fine and coarse particles. The linear model was used to determine the contributions of time and granulometry, as well as their interaction. The effects of time and granulometry were highly significant for the TPC response, while their interaction did not affect it significantly. The effect of time was constant (linear regression with angular coefficient = 0.55 and intercept 9.48), depending on the starting granulometry, which showed an additive effect (8.60 to the intercept).

Although the interaction between the two factors was not statistically significant, the 212 µm granulometry negatively affected the angular coefficient of the regression of the TPC extracted over time (−0.016), suggesting that US plays a role in reducing the size of the biggest particles.

### 3.3. Conventional Extractions

#### 3.3.1. Silent Conditions

In order to investigate the effectiveness of US, the main tests conducted for l-UAE were reproduced under silent conditions. It was therefore possible to separate the role of cavitation from that of temperature, and to quantify it by means of percentage TPC variation, as reported in Table 5.

Generally, we can state that acoustic cavitation achieved better results for every tested condition, with maximum extraction intensification being found for the <1000 µm fraction.

#### 3.3.2. Benchmark Comparison

According to the extraction yields observed so far, temperature appeared to play a significant role in the process outcome. For this reason, the TPC of an aqueous extract under reflux conditions was evaluated. Furthermore, the protocol was repeated twice on the same matrix in order to achieve exhaustive extraction. The partial and global yields are reported in Table 6.

The TPC trend shows that high-temperature conditions are not favorable for polyphenols extraction in an aqueous media, even in a multistep approach.

In terms of yield, hydroalcoholic solutions are commonly considered a reference solvent for conventional approaches to polyphenols recovery from vegetal matrices. An optimized protocol [22] was therefore reproduced for use as a conventional hydroalcoholic benchmark (see Table 6), although this test cannot be considered strictly comparable because of the variation in the solvent polarity.

The <1000 µm fraction led to a TPC recovery that was roughly comparable to that of l-UAE, while smaller particles provided a great increase in the polyphenols concentration in the final product.

### 3.4. HPLC-DAD, UPLC-ESI-MS/MS Analyses, and OPC-Content Determination

The results from the HPLC-DAD analyses of l-UAE (H_2_O, cup-horn, 45 min, 45 °C) and the conventional hydroalcoholic benchmark (EtOH/H_2_O 60:40, 23 min, 95 °C, reflux) are reported in Table A3 and Table A4, using the method for polyphenols (Pol) and catechins (Cat), respectively. The listed compounds were identified by comparing the retention times and UV spectra with the external standards (ST), when possible, or by comparing the extracted UV spectrum with those available in the literature (UV). When matched with an external standard between those that were screened (see Section 2.1), the compounds were quantified using a calibration curve, and the amounts, expressed as mg/g DE, are indicated in the Table A3. When identified by the UV spectra, the compounds were not quantified. For the quercetin derivatives, such as 3-glucuronide and 3-rutinoside (rutin), the aglycone calibration curve was used for quantification, which was expressed as quercetin equivalents mg/g DE (Table A3). The retention times (RT), λ max, λ for eq., equation curves, linearity ranges, R^2^, LOD (limit of detection), and LOQ (limit of quantification) for all of the identified standard compounds (ST) are listed in Table A5.

**Figure A3:**
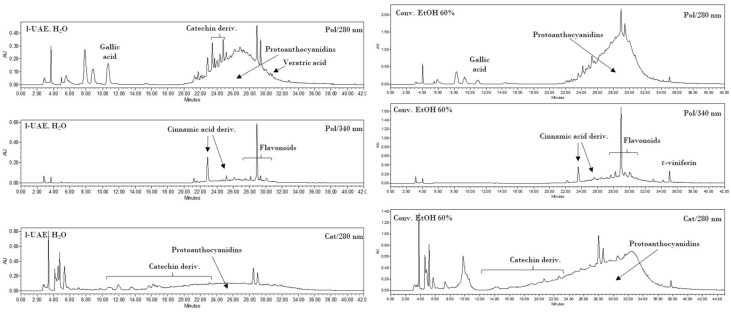
HPLC-DAD chromatograms of l-UAE (H_2_O, C-horn, 45 min, 45 °C; first and second on the left, 280 and 340 nm, respectively) and conventional hydroalcoholic extracts (EtOH/H_2_O 60:40, 23 min, 95 °C, reflux; first and second on the right, 280 and 340 nm, respectively) recorded with method Pol (HPLC method Pol) or recorded with method Cat at 280 nm (third on the left, l-UAE, and third on the right, conv.)

In Figure A3, chromatograms at 280 and 340 nm for Pol and 280 nm for Cat are reported for both the optimized l-UAE (H_2_O, cup-horn, 45 min, 45 °C) and the conventional hydroalcoholic benchmark (EtOH/H_2_O 60:40, 23 min, 95 °C, reflux). The principal compounds and polyphenol classes are highlighted in correspondence to the peaks indicated in Table A3 and Table A4. As shown in the chromatograms, correct identification and/or quantification was not possible in some cases because of the broad peaks from 21 to 32 min (280/Pol) and 18 to 36 min (280/Cat), because of the presence of OPCs.

**Figure 4:**
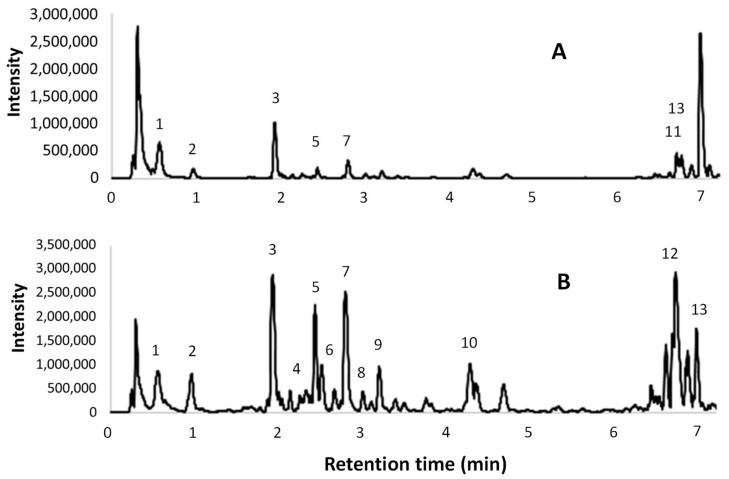
Electrospray ionization (ESI)^-^ total ion chromatograms obtained from UPLC-ESI-MS/MS analyses; (**A**) l-UAE (water, cup-horn, 45 min, 45 °C); (**B**) conventional hydroalcoholic extracts (EtOH/H_2_O 60:40, 23 min, 95 °C, reflux).

The UPLC-ESI-MS/MS chromatograms of the same two extracts are shown in Figure 4. A tentative identification of the compounds that are present in the extracts, based on [M – H]^–^ and MS fragments, is reported in Table 7. The peak numbers are reported in both the table and in the ESI(^–^) total ion chromatograms.

Because of the extended quantity of the catechin-derived oligomeric compounds present in the tested extracts, the OPC content for both l-UAE and the conventional hydroalcoholic benchmark was determined using the butanol-HCl method, with procyanidin B2 as the reference compound. The OPC content in l-UAE (H_2_O, cup horn, 45 min, 45 °C) was 30.5 mg/g on the dried extract (DE), while for the conventional hydroalcoholic extracts (EtOH/H_2_O 60:40, 23 min, 95 °C, reflux), an amount of 47.6 mg/g DE was found.

### 3.5. Ultrasound-Assisted Extraction: Scale-Up (S-UAE)

Using the l-UAE results as a foundation, it was possible to evaluate the transposition of grape-stalk extraction to a kilo-scale protocol. With this aim, the optimized lab-scale parameters were adjusted to a recirculating US flow system, for which the vegetal matrix (<1000 µm) was mixed with water at 45 °C. The main process parameters are summarized in Table 8.

Reproducing the experimental method adopted so far, periodic sampling allowed for information about S-UAE efficacy to be gathered via TPC quantification (Table 9). In consideration of the recycling set-up, the time range was extended to 120 min so as to deepen the process characterization.

**Figure 5:**
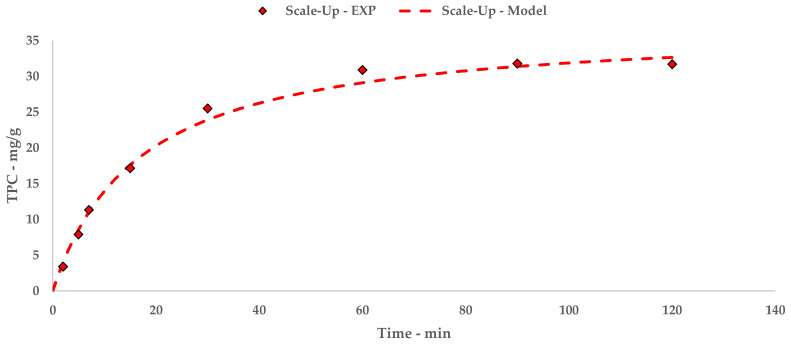
S-UAE: experimental values and Peleg model.

**Figure A4:**
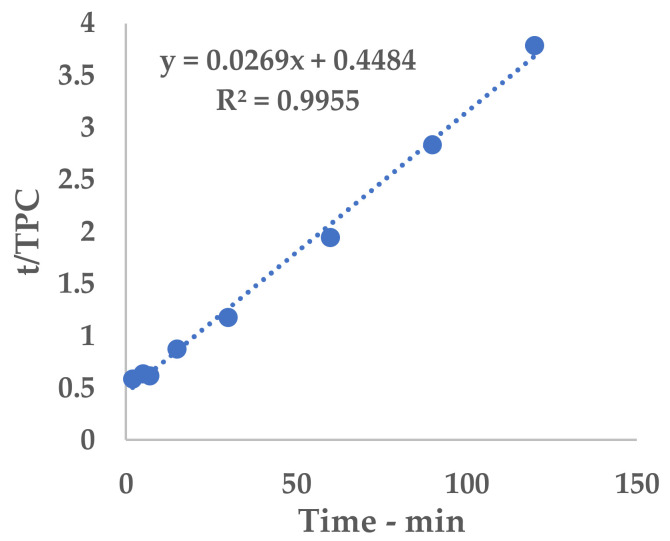
Linearization, S-UAE.

The polyphenolic recovery appears to be consistent with the previous studies, achieving TPC with similar orders of magnitude. Similar to l-UAE, these values were exploited to extrapolate a hyperbolic equation that described the kinetic curve of the flow system (Equation (9) and Figure 5). The linearization and related table are reported in Appendix A (Table A6 and Figure A4).

(8) $$C\left(t\right)=\frac{t}{0.4484+0.0269\;t}$$

The trend and general yield depicted by the kinetic study for S-UAE appear to be comparable to the l-UAE investigations, for which the hyperbolic model coherently matches the experimental points.

The increased processing volumes mean that a suitable approach that can handle the production stream is required. Hence, NF was used to concentrate the crude extract after S-UAE, removing the maximum amount of water possible.

Dedicated screening was carried out on a pilot membrane skid. The retentate was continuously re-injected into the feed tank during the process, while the aqueous permeate was directed to a separate vessel. The sampling times and relative data analysis are reported in Table 10.

After 50 min of the NF process, with an adequate pressure adjustment, the crude liquid was reduced to 20 L, resulting in a global extract concentration of 355.91%. The NF protocol was continued until the onset of particle precipitation.

The isolated final product, obtained after the concentration process, was characterized by means of TPC and DPPH^•^ scavenging activity assays. The scavenging effect of the selected grape-stalk extracts was screened to evaluate their DPPH^•^ inhibition percentage (I%) at a variety of concentrations, and it was expressed as the mean value of the half maximal effective concentration (EC_50_, µg/mL) ± standard deviation (SD). The Trolox^®^ standard showed an EC_50_ value of 3.94 ± 0.50 µg/mL. The trolox^®^ equivalents (TE), expressed as µmol/g of extract (EXT), were calculated based on EC_50_. The results pre- and post-NF are summarized in Table 11, and are compared with the C-horn data.

It appears that the TPC content was not significantly affected, when compared with the S-UAE sample at 120 min (32.07 mg/g vs. 32.60 mg/g). This evidence supports the idea that NF’s predominant effect is water removal, confirming that the overall polyphenolic losses are negligible.

The antioxidant power, evaluated by means of DPPH^•^, shows that a high activity is maintained by the S-UAE approach. Furthermore, similarly to TPC, NF does not compromise the overall activity.

## 4. Discussion

### 4.1. Ultrasound-Assisted Extraction: Lab-Scale (l-UAE)

UAE was the extraction technique selected to recover the antioxidant compounds from the residues of the grape stalks. l-UAE was investigated in the form of an I-horn and a C-horn. The peculiarities of each system were thus observed during the tests, and the aspects that provided the best performance were investigated. For this purpose, TPC evaluation was adopted as a control parameter for kinetic definition, and the Peleg model was used over a time range of 45 min.

Granulometry distribution provided the possibility of using a selected matrix fraction. As reported in Section 3.1, a >1000 µm mesh is prone to agglomeration, and hence dramatically hampers the processability and overall mass transfer of the suspension (inhomogeneous and highly viscous system). UAE that was performed on particle sizes of less than 1000 µm allowed the features of cavitation to be properly exploited, while still being able to treat a bulk quantity of the starting material. The screenings reported in Section 3.2.1 and Section 3.2.2 focused on <1000 µm granulometries.

I-horn was first used to screen the RT and 45 °C extractions (See Table 2). As expected, it was immediately clear that temperature plays an important role, as it increased the overall TPC, independently of the process time. In particular, it was observed that effective diffusivity (D) depends on temperature, according to a first-order rate process that is generally described by the Arrhenius equation,

(9) $$D=D_{0\;}e_{\;}^{-\frac{E_{a}}{RT}}$$

where D_0_ is the pre-exponential factor (m^2^/sec), E_a_ is the activation energy (kJ/mol), R is the gas constant (8.3145 × 10^−3^ kJ/mol/K), and T is the absolute temperature (K).

The selectivity of I-horn UAE, however, appeared not to be affected by heat, but rather by extraction duration. Nevertheless, this behaviour was not particularly significant, as the polyphenols recovery appeared to consistently slow after approximately 30 min. The extrapolation of a kinetic model, using the Peleg method, allowed for the physical trend of the process to be described by quantifying the peculiar parameters.

The kinetic constants, k_1_ and k_2_, could be used to calculate the extraction rates and steady state TPC yields for a single stage of equilibrium (see Equations (2) and (3)). The results are reported in Table 12. A comparison of B_0_ confirms that the 45 °C UAE almost doubled the process speed, against a strictly analogous Y_S_.

Tests performed with the I-horn revealed that a temperature of 45 °C can drastically increase the extraction rate, while still remaining in the US working-temperature range [30]. For this reason, RT was not considered for the kinetic screening of the C-horn, and it was found that the higher temperature influenced the selectivity of this system (see Table 2). In fact, it appeared to be higher than that of the I-horn (average 9.44% vs. 6.72%) and gave an interesting increase in TPC during the extraction process, as well as the best metabolite recovery so far. Furthermore, it is possible to evaluate the extraction trends from the kinetic parameters extrapolated by the related Peleg equation (Equation (8)). It is possible to observe the extraction rate enhancement at 45 °C, correlated with the US device, in Table 12. Concerning Y_S_, the C-horn registered a very slight increase, reporting 35.09 mg/g of GAE against the 34.13 mg/g of the I-horn.

According to the collected evidence, C-horn proved itself to be the more proficient US device for the extraction of polyphenolic compounds from grape stalks. Furthermore, kinetic profiles confirmed a fast extraction trend, simplifying the trade-off between process time and final TPC yield.

#### Effect of Granulometry

So far, the investigated extractions have provided details and models for stalks with granulometries <1000 µm. Coarser particles were excluded from the investigation in order to minimize the clustering phenomena during UAE. It is assumed that the loss of active compounds associated with size selection is limited, mainly because of the fibrous nature of the >1000 µm fraction. This assumption is supported by the literature, which has shown that the metabolite composition differs with granulometry [29,31].

In addition, the effect of smaller particles on UAE efficacy was taken into consideration, and the 212 µm fraction was processed (2.7% of the whole matrix). The effect of comminution on the overall process outcome was evaluated. The percentage differences reported in Table 3 highlight how the particle-size effect is consistent under RT conditions, while heat considerably attenuates the yield enhancement. It is worth stating that the applied US technique can partially affect polyphenols recovery. This is probably because of the stronger size-reduction effect of the I-horn, which is due to a direct probe-matrix interaction.

A time-dependent trend with the I-horn at RT was explored for coarser and finer particle fractions (see Table 4). The linear model (two-way ANOVA) was used to determine the contributions of time and granulometry, as well as their interaction (Table A7). The effects of time and granulometry were highly significant for the TPC response (*p* < 0.001), while their interaction did not affect it significantly (0.1 < *p* < 1). The effect of time was constant (linear regression with angular coefficient = 0.55 and intercept 9.48) and depended on the starting granulometry, which showed an additive effect (8.60 to the intercept). Although the interaction between the two factors was not statistically significant, the 212 µm granulometry negatively affected the angular coefficient of the regression of the TPC extracted over time (−0.016), suggesting that US plays a role in the size reduction of the biggest particles, as has already been reported in the literature [32].

The screenings carried out in Section 3.2.3. revealed that the smaller granulometry of the starting material can appreciably affect final TPC working under mild conditions (RT, either silent or UAE), while an almost negligible effect was observed under UAE at 45 °C.

### 4.2. Conventional Extractions

#### 4.2.1. Silent Conditions

To corroborate the suitability of US for polyphenols recovery from grape stalks, the optimized l-UAE protocols were reproduced under silent conditions. In order to emphasize the role of the technology, a number of different set-ups, temperatures, and granulometries were considered, as shown in Table 5. It is worth noting that UAE enhanced TPC under every experimental condition. A maximum was reached (Silent/l-UAE yield 36.57%) when coarser particles were extracted at RT. This result can further confirm the intensification achieved by means of cavitation, which is particularly marked when the contribution of heat is removed and when mass transfer is hindered by a low matrix surface area (<1000 µm fraction). Further strengthening the case for the best performing UAE protocol so far (C-horn, <1000 µm fraction, 45 °C), the silent test resulted in an interesting 30.16% yield reduction.

To better compare the effects of the extraction conditions, such as time, temperature, US, horn type, and matrix granulometry, three-way ANOVA was performed on the TPC values reported in Table 3; Table 5. The first factor described the different times and temperatures used for the extractions (three levels, 45 min/45 °C for C-horn, 30 min/RT and 30 min/45 °C for I-horn), the second indicated the different starting granulometries (two levels, 212 and <1000 µm), and the third showed the influence of US irradiation at the same time and temperature as the silent-condition runs (two levels, no US or US; Table 13). The extraction conditions, the presence of US, and the granulometry were all highly significant factors that influenced the TPC yields (one-way, *p* < 0.001, Table A8), as was the interaction between granulometry and US (two-ways, *p* < 0.001). Indeed, a bigger increase in TPC values was evidenced when US was used with a granulometry <1000 µm, because of its well-documented crushing effects [32]. The interaction between the extraction conditions and US irradiation was related to the different times and temperatures at which they were applied (two-way, 0.01 < *p* < 0.05). The interactions between US and granulometry (two-ways, *p* > 0.05), and between extraction conditions, US, and granulometry were not statistically significant (three-ways, *p* > 0.05).

Multiple comparisons were made one factor at a time, using Tukey’s HSD post-hoc test with the Bonferroni correction, a confidence level of 0.95, and a significance level α = 0.05. The confidence level and *p*-value adjustments with the Bonferroni method were based on the number of tests and estimates performed (two or three, based on the factor considered). In particular, in Table 13, multiple comparisons between the three levels of the extraction conditions at fixed levels of granulometry and the applied technique (US or silent) were presented.

Starting from the 212 µm granulometry without US, no significant differences in TPC yields were found under the different extraction conditions (time and temperature, column 2 in Table 13). Under US irradiation, the best results were obtained for I-horn/30 min/RT and C-horn/45 min/45 °C, which were both significantly different to I-horn/30 min/45 °C (column 3, Table 13). This last run had a lower value, which was probably due to the degradation of polyphenols at higher temperatures and times using the immersion horn.

Starting from a granulometry <1000 µm, for which US was not applied, significant differences in the TPC yields were found between the extraction conditions at 45 °C (C-horn/45 min/45 °C, I-horn/30 min/45 °C) and the one at RT (I-horn/30 min/RT; column 4, Table 13). The temperature was a key factor for increasing polyphenols extraction in the absence of US. C-horn/45 min/45 °C gave the best results when using US irradiation on this higher granulometry, and was significantly different to I-horn/30 min/45 °C and I-horn/30 min/RT (column 5, Table 13). This corroborated our choice to scale-up US using <1000 granulometry.

The granulometry was always a significant factor for TPC values when US was not applied, while in the presence of US, it was significant only for treatment at Rt (I-horn/30 min/RT; not reported in Table 13).

US irradiation gave significant improvements in TPC yields under all conditions (time and temperature) and starting granulometries, except for I-horn/30 min/45 °C on the 212 µm granulometry, because of a possible degradative effect (not reported in Table 13).

#### 4.2.2. Benchmark Comparison

Heat was found to be a relevant parameter that affected yield enhancement when reviewing US suitability for grape-stalk extraction. A dedicated experiment was therefore carried out to explore the influence of higher working temperatures on TPC in aqueous media. Given that the requirement was to investigate conditions outside of the US-applicability range, an extensive conventional reflux approach was chosen. The protocol was repeated twice on the same matrix, with the aims of achieving exhaustive extraction and of verifying how polyphenols recovery can be affected (see Table 6). Surprisingly, the first extraction gave a very low concentration of polyphenols, despite the high dry extract weight, and showed a very low selectivity due to a significant concentration of interfering co-extract compounds. The second step achieved a better result, but still maintained a very low cumulative recovery of 15.44 mg/g. The TPC trend suggests that high-temperature conditions are not favorable for polyphenols isolation in water media, even for prolonged treatment times, meaning that fast, low-temperature approaches are preferred.

The aim of this work is to propose a sustainable and cost-effective extraction process for grape stalks that can be easily scaled-up to an industrial scale. In line with these aims, water was selected as the ideal solvent, despite the fact that hydroalcoholic solutions are commonly considered as a reference in terms of outcomes. Hence, a conventional hydroalcoholic protocol [22] was performed as a benchmark for the sake of comparison (see Table 6), although this test is not strictly comparable because of the variation in solvent polarity. The reported TPC for the <1000 µm fraction (37.04 mg/g) showed a very similar value to l-UAE, namely 34.13 mg/g and 35.09 mg/g for I-horn and C-horn at 45 °C, respectively. On the other hand, the smaller particle size led to a dramatic yield increase. However, this was counterbalanced by the minimal quantity of the 212 µm fraction available in the roughly milled stalks (2.7%). Another aspect to be considered is that particle differentiation is partially a consequence of the different physical characteristics (i.e., elasticity and fibrousness) of the different plant sections (stems, tendrils, etc.). Various plant structures have differing metabolite contents, meaning that complete matrix comminution may not necessarily result in a consistent yield enhancement for all fractions. Furthermore, the extreme milling conditions required to obtain very fine particles can lead to local heat generation in the biomass, thus promoting degradation phenomena, and reducing the biological and antioxidant activity.

In addition to the above, severe extraction temperatures, together with dedicated and harsher milling treatments, considerably decrease the cost-efficacy and sustainability of the overall process. These factors, concurrently with ethanol costs and managing issues (i.e., taxes and plant-security requirements) may have a large impact on the net profit of processing a valuable matrix, such as grape stalks.

### 4.3. HPLC-DAD, UPLC-ESI-MS/MS Analyses, OPC-Content Determination

The principal compounds identified in the l-UAE (H_2_O, cup-horn, 45 min, 45 °C) and conventional extracts (EtOH/H_2_O 60:40, 23 min, 95 °C, reflux), using the Pol HPLC method at 280 nm, were gallic and veratric acid, together with catechin derivatives (OPCs). On the other hand, using the Pol method at 340 nm, cinnamic acid derivatives; stilbenoids, such as ε-viniferin; and flavonoids, such as isorhamnetin; quercetin, and kaempferol derivatives were detected (Table A6 and Table A7, Figure A4). The 340 nm wavelength was more selective, giving a better baseline despite the broad peak due to the OPCs. Therefore, the quantification of some flavonoids was possible. The amount of quercetin-3-glucuronide was 0.905 vs. 7.34 mg/g DE for the l-UAE and conventional extracts, respectively, while the rutin content was 1.09 mg/g DE in the l-UAE extract (both expressed as quercetin equivalents). The hydroalcoholic extract was richer in quercetin derivatives because of their better solubility in EtOH than in H_2_O. For the same reason, ε-viniferin (2.89 mg/g DE) and a kaempferol (not quantified) derivative were only found in this extract. On the other hand, the l-UAE water extract showed a higher gallic-acid content (5.70 vs. 3.86 mg/g DE in the l-UAE and conventional extracts, respectively).

The Cat HPLC method at 280 nm (Table A6 and Table A7, Figure A4), set up for the separation of catechin mixtures (see Section 2.1), detected a higher content of these metabolites in the conventional extract ((+)-catechin content of 5.58 vs. 3.98 mg/g DE for l-UAE and conventional extracts, respectively). Nevertheless, for conventional hydroalcoholic extracts, the broad peak of the OPCs precluded the correct identification and/or quantification of compounds between 20 and 36 min.

UPLC-ESI-MS/MS chromatograms (Figure 4) were used to support the identification of some compounds found in the HPLC-DAD analyses. The tentative identifications in Table 7 confirmed the presence of several procyanidin oligomers (peaks 4, 6, and 8–10) ([M-H]^-^ 1153 (*m/z*) and MS fragments 865, 577, and 289 (*m/z*)), and a procyanidin dimer (5) ([M – H]^–^ 577 (*m/z*) and MS fragments 289 and 245 (*m/z*)), in particular in the conventional hydroalcoholic extract. Gallic acid (2), (+)-catechin (7), and isorhamnetin (3) were confirmed. Citric acid was found in the [M – H]^–^ 191 (*m/z*) and MS fragments 11, 129, and 85/87 (*m/z*). The quercetin derivatives were identified as rutin (quercetin-3-rutinoside) with [M – H]^–^ 609 (*m/z*) and MS fragments 463, 301 (*m/z*), and quercetin-3-glucuronide with [M – H]^–^ 477 (*m/z*) and MS fragments 463 and 301 (*m/z*). Finally, kaempferol-3-O-glucoside-7-rhamnoside was found with [M – H]^–^ 593 (*m/z*) and MS fragments 447 and 285 (*m/z*).

The OPC content was in agreement with the HPLC findings, as it was higher for the conventional hydroalcoholic extract than the l-UAE extract in water.

### 4.4. Ultrasound-Assisted Extraction: Scale-Up (S-UAE)

In light of the l-UAE screenings, an experimental transposition to a kilo-scale process was explored. A flow configuration was chosen as a suitable solution in order to effectively handle the increase in processing volumes. The move from the batch system means that it is crucial to find a connection point with results that defined the C-horn as the best performing device. A flow-through cell (Biopush, Weber Ultrasonics) was identified as a suitable answer to the case-study. Nevertheless, as the batch-flow transposition is not geometric, it was necessary to adapt some parameters for processability. In particular, a recycling protocol, with a loop configuration set-up, was applied, providing a cumulative residence time (τ_R_) that is comparable to that of l-UAE (see recycling time, Table 8).

Just as for the l-UAE tests, the TPC that was recovered at different time points was used to extrapolate a kinetic model, providing an equation and relative constants. The kinetic curve can be compared with the l-UAE ones with the appropriate approximations (Figure 5). The initial S-UAE curve slope is less steep than those of the batch lab-scale systems. This is likely due to the recycling approach; the system can process 15 L of suspension at one time. This is then pumped inside the tank containing the remaining 45 L. The treated matrix is therefore continuously merged with the previous fraction, improving the S/L contact, but partially delaying sonication. Moreover, the flow set-up may lead to less effective cavitation events, compared with the closed lab-scale devices, because of the onset of a turbulent motion.

**Figure 6:**
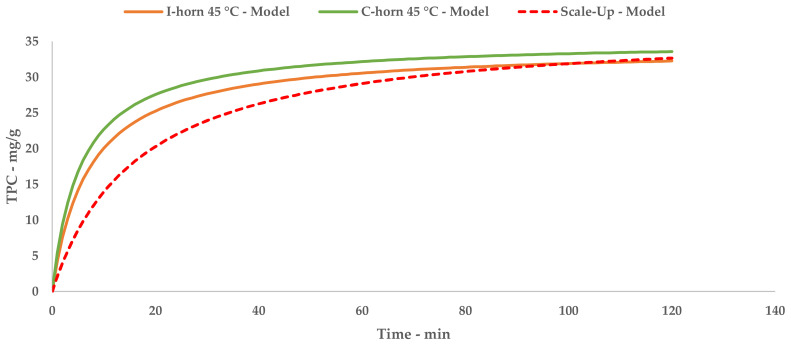
Lab-scale/scale-up extraction curve comparison at 45 °C.

A direct comparison of the kinetic parameters (Figure 6) shows how the flow system possesses an extraction rate that is comparable to the I-horn RT protocol (2.23 mg/g min vs. 2.77 mg/g min), and conversely displays the best Y_S_ enhancement (37.7 mg/g).

### 4.5. Nanofiltration (NF) Concentration

The extraction scale-up entailed a drastic increase in processed volumes, meaning that it was crucial to identify a suitable approach in order to isolate the final product. Membrane filtration seemed to be a convenient technique to reduce the water mass and concentrate the polyphenolic fraction. In particular, NF was able to efficiently separate the crude extract in a metabolite-rich retentate stream, with negligible dispersion. The resulting water was recovered as a permeate for potential recycling.

**Figure 7:**
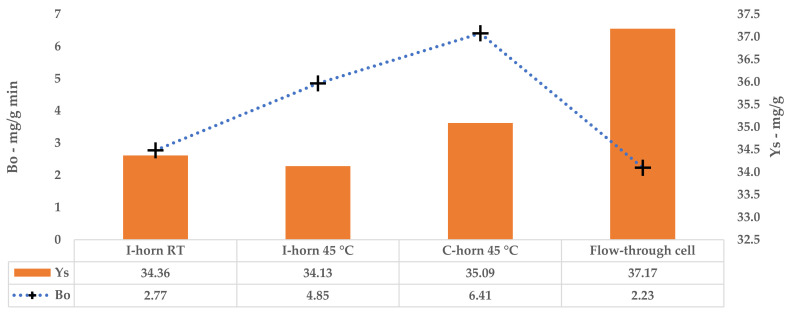
Lab-scale/scale-up kinetic-parameter comparison.

During the volume contraction, expressed as ResVol, it was possible to observe a constant GConc increase (Figure 7, blue line), and a final extract concentration of 355.91% was achieved after 50 min. Approximately 80 L of aqueous permeate was removed, cutting down the overall solution amount by 80%. The InstConc behaviour allowed us to outline how NF efficacy is strictly dependent on the progressive extract concentration. In particular, after a ResVol of 70 L, the membrane performance decreased and reached a minimum at 50 and 40 L (Figure 7, orange area). To balance the phenomenon (detectable as a pressure alteration during the process), the counter-pressure was raised from 10 to 15 bar. The new set-up resulted in a sudden InstConc improvement that translated into a GConc slope increase as well. NF reached its conclusion at the first sign of particle precipitation as the saturation limits were attained.

**Figure 8:**
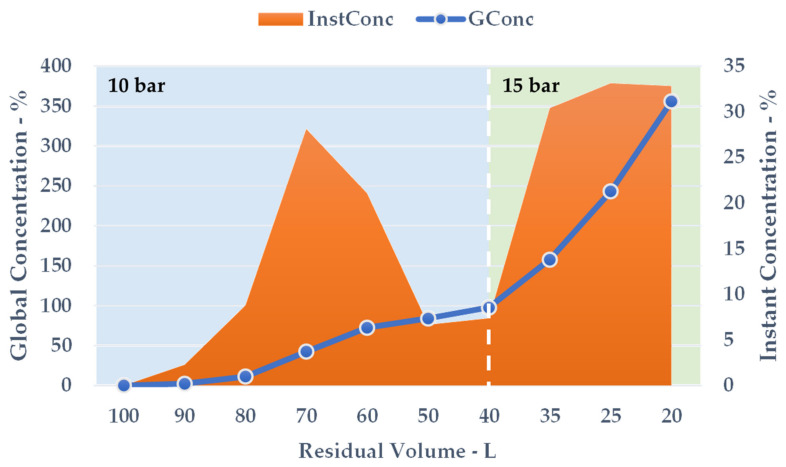
NF concentration trend, counter-pressures reported.

**Figure 9:**
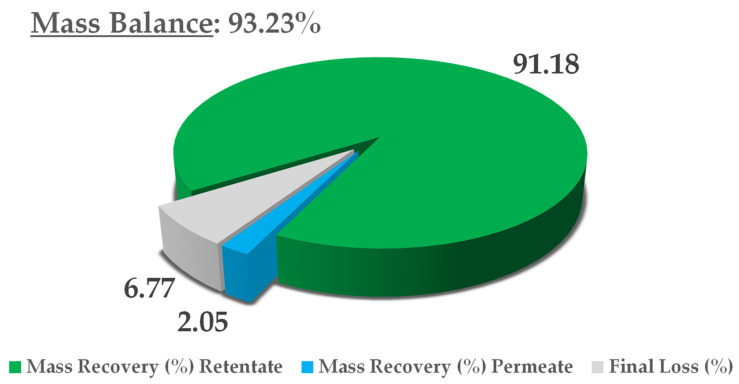
Mass-balance report.

The final thoughts on this process concern mass balance, in consideration of the dry yield of all NF streams, namely, the retentate and permeate. The extract content at minute 0 was adopted as a reference, and the theoretical final concentration was derived from a simple volume contraction. The results are reported in Figure 8. In light of an overall mass balance of 93.23%, the main extract fraction was recovered in the retentate (91.18%), with a negligible residue in the permeate (2.05%). Considering that this is a pilot process, we can say that limited loss was registered (6.77%), most likely because of the non-exhaustive recovery from the utilities used (i.e., pump and valves). See Figure 9 for mass balance resume.

To the best of our knowledge, this is the first time that NF has been applied to concentrate grape-stalk extract, reducing the impact of water evaporation on the process. The closest example applied ultra-e nano-filtration to the hydroalcoholic extract of grape pomace [33]. In the work of Zagklis et al., the mandatory removal of ethanol before membrane use, together with a previous application of ultrafiltration, negatively affected the final polyphenolic content. Thus, a general polyphenols recovery of 65.2% was determined, with respect to a 160.7% product concentration (vs. 355.91%). A larger MWCO (470 Da vs. 300 Da) can partially explain the different outcomes. Consequently, according to results obtained in this work, it is possible to reduce the general losses exploiting NF with the purpose of only solvent removal, thus avoiding product fractionation. The application of simple water allowed for the direct application of membranes, enhancing the overall sustainability of the process.

## 5. Conclusions

This piece of work investigated the UAE in water of polyphenols from grape-stalk residues. Lab-scale screenings were performed with both I-horn and C-horn at RT and 45 °C on two different matrix granulometries (<1000 µm and 212 µm). Physical models of the extraction trends could be extrapolated from the kinetic studies on the base of TPC yields. C-horn was the best performing lab-scale device. The effect of the starting material size, the comminution effect of US, and the extraction temperature have been discussed [32]. TPC yields showed how the extraction temperature and acoustic cavitation can mainly override the granulometry influence.

The extract obtained with the best performing protocol has been characterized by means of HPLC-DAD, UPLC-ESI-MS/MS, and OPC quantification. Two different analytical methods for HPLC revealed interesting contents in the hydroxycinnamic and hydroxybenzoic acids (i.e., gallic and veratric acids), with a large variety of catechin derivatives, assigned to OPC and OPC fragmentation, being observed. This last consideration is supported by qualitative UPLC and colorimetric OPC quantification.

The results achieved on the lab scale made it possible to evaluate scale-up in a US recirculating flow system. We have proposed a kilo-scale extraction strategy, in which dedicated work-up procedures entail pre-industrial facilities, such as decanter and bag-filter units. Moreover, the increase in the quantity of starting material also requires a significant amount of water. We have therefore proposed using an NF protocol to deal with the large volumes in a sustainable way. The pilot NF skid concentrated the crude liquid extract by up to 355.91%, saving 80% of the water for possible reuse in the extraction process. TPC and antioxidant power evaluations confirmed that UAE can be successfully transposed from the gram- to kilo-scales, with limited losses in terms of yield and activity.

## Figures and Tables

**Table 1:** Composition and granulometry of grape stalks.

Composition			Granulometry	
Water	Ashes	Organic	Mesh	Ratio
(% *w/w*)			(µm)	(% *w/w*)
8.92	4.46	86.63	>1000	46.7
			1000	40
			500	10.7
			212	2.7

**Table 2:** Dry extract (DE) and total phenolic content (TPC) yields for ultrasound-assisted extraction: lab-scale (l-UAE).

Extr. Time (min)	I-horn RT			I-horn 45 °C			C-horn 45 °C		
	DE ^a^ (mg/g)	TPC ^b^ (mg/g)	Select. (%)	DE ^a^ (mg/g)	TPC ^b^ (mg/g)	Select. (%)	DE ^a^ (mg/g)	TPC ^b^ (mg/g)	Select. (%)
5	180.07	10.86	6.03	258.92	15.7	6.06	180.07	16.03	8.90
8	238.07	14.22	5.97	281.55	17.13	6.08	238.07	21.71	9.12
10	248.24	15.27	6.15	287.26	17.07	5.94	248.24	22.95	9.25
12	261.62	15.76	6.02	304.5	22.529	7.40	261.62	24.42	9.33
15	262.97	17.12	6.51	372.26	26.32	7.07	262.97	26.25	9.98
30	287.11	26.03	9.07	377.32	28.24	7.08	287.11	28.24	9.84
45	330.99	26.59	8.03	399.87	29.71	7.43	330.99	31.89	9.63

^a^ Expressed over dry matrix (DM); ^b^ Expressed as gallic acid equivalents (GAE) over DM.

**Table 3:** Granulometry effect on l-UAE.

l-UAE	*t*	*T*	Granulometry	TPC ^a^	Difference
	(min)	(°C)	(µm)	(mg/g)	(%)
I-horn	30	RT	<1000	26.03	30.93
			212	34.08	
		45	<1000	28.24	3.15
			212	29.11	
C-horn	45	45	<1000	31.89	6.62
			212	34.00	

Three-way ANOVA, three factors, 3×2×2 levels; ^a^ Expressed as average values of GAE over DM (SE 0.81, confidence level 0.95).

**Table 4:** Effect of starting material granulometry on extraction time for I-horn at room temperature (RT).

t	Granulometry	TPC ^a^
(min)	(µm)	(mg/g)
8	<1000	14.22
	212	22.51
15	<1000	17.12
	212	25.77
30	<1000	26.03
	212	34.08

Two-way ANOVA, two factors, 3 × 2 levels; ^a^ Expressed as average values of GAE over DM (residual standard errors (SE) 1.188 on 14 df, multiple R^2^ 0.9744).

**Table 5:** Silent conditions comparison.

l-UAE Control	*t*	*T*	Granulometry	Silent TPC ^a^	Yield Drop ^b^
	(min)	(°C)	(µm)	(mg/g)	(%)
C-horn	45	45	<1000	24.50	30.16
			212	27.09	25.51
I-horn	30	RT	<1000	19.06	36.57
			212	26.11	30.52
	30	45	<1000	22.17	20.57
			212	26.81	26.30

Three-way ANOVA, three factors, 3×2×2 levels; ^a^ Expressed as average values of GAE over DM (SE 0.81, confidence level 0.95); **^b^** Compared to the UAE control.

**Table 6:** Dry extract (DE) and TPC yields for conventional extractions.

Extraction Solvent	*t* (min)	*T* (°C)	Granulometry (µm)	DE ^a^ (mg/g)	TPC ^b^
					(mg/g)
H_2_O	180 ^c^	100	<1000	637.4 (345.31/292.06) ^d^	15.44 (1.83/13.61) ^d^
EtOH/H_2_O 60:40	23	95	<1000	348.46	37.04
EtOH/H_2_O 60:40	23	95	212	266.47	63.60

^a^ Expressed over DM; ^b^ Expressed as average value of GAE over DM (SE 0.63, confidence level 0.95); ^c^ Single step length; ^d^ Results expressed as: total yield of two steps (partial yields of each step).

**Table 7:** Tentative identification of the compounds in the samples by UPLC-ESI(^–^)-MS/MS.

Peak	Rt (min)	[M – H]^–^	MS Fragments	Tentatively Identified Compounds
1	0.56	191	11, 129, 85/87	Citric acid
2	1.02	169	125	Gallic acid
3	1.92	315	153	Isorhamnetin
4	2.35	1153	865, 577, 289	Procyanidin oligomer
5	2.43	577	289, 245	Procyanidin dimer
6	2.51	1153	865, 577, 289	Procyanidin oligomer
7	2.79	289	245, 205	(+)-Catechin
8	3.01	1153	865, 577, 289	Procyanidin oligomer
9	3.27	1153	865, 577, 289	Procyanidin oligomer
10	4.30	1153	865, 577, 289	Procyanidin oligomer
11	6.73	609	463, 301	Rutin (quercetin-3-rutinoside)
12	6.75	477	301, 178, 151	Quercetin-3-glucuronide
13	6.87	593	447, 285	Kaempferol-3-*O*-glucoside-7-rhamnoside

**Table 8:** Flow transposition main parameters.

Flow Rate	Reactor Vol.	τ_R_	Process Vol.	Recycle Time ^a^
(L/min)	(L)	(min)	(L)	(min)
30	15	2	60	4

^a^ Time required to treat the entire matrix for 1 min.

**Table 9:** TPC yields for scale up (S)-UAE.

Extr. Time (min)	TPC ^a^ (mg/g)
2	3.41
5	7.89
8	11.32
15	17.16
30	25.51
60	30.88
90	32.71
120	32.60

^a^ Expressed as GAE over the dry extracted matrix.

**Table 10:** Nanofiltration (NF) screening.

Time	Residual Volume ^a^	Global Conc. ^b^	Counter-Pressure	Instant Conc. ^b^
(min)	(L)	(%)	(bar)	(%)
0	100	0	10	0
1	90	2.28	10	2.28
6	80	11.27	10	8.79
12	70	42.57	10	28.13
18	60	72.60	10	21.06
24	50	84.10	10	6.67
30	40	97.70	10	7.39
38	35	157.81	15	30.40
45	25	243.21	15	33.13
50	20	355.91	15	32.84

^a^ Progressive feed reduction; ^b^ Expressed on dry residues.

**Table 11:** TPC and antioxidant activity of the various extracts, expressed as DPPH^•^ EC_50_ values and as TEAC.

Sample	TPC ^a^ (mg/g DM)	DPPH EC_50_^b^ (µg/mL)	TEAC ^c^ (µmol/g)
S-UAE (Pre-NF)	32.60	104.8 ± 30.6	150.2 ± 43.9
S-UAE (Post-NF)	32.07	123.9 ± 27.4	127.1 ± 28.1
C-horn ^d^	31.89	117.1 ± 33.6	134.4 ± 38.6
Trolox^®^	-	3.94 ± 0.50	-

^a^ Expressed as GAE over the dry extracted matrix; ^b^ mean value (± standard deviation (SD)) of the maximal effective concentration of compound/extract necessary to decrease the initial concentration of DPPH• to 50% at equilibrium; ^c^ expressed as µmol Trolox^®^ equivalents (TE) per g of extract (EXT); ^d^ optimized l-UAE (H_2_O, 45 min, 45 °C).

**Table 12:** Kinetic parameters of l-UAE.

l-UAE	Temperature	B_0_^a^ (mg/g min)	Y_S_^b^ (mg/g)
I-horn	RT	2.77	34.36
	45 °C	4.85	34.13
C-horn	45 °C	6.41	35.09

^a^ starting extraction rate; ^b^ highest extraction yield.

**Table 13:** TPC means (mg/g DM) obtained in different extraction conditions at different granulometries, in the presence or not of US irradiation, and multiple comparisons of the fixed granulometry and techniques applied (US).

Extraction Conditions (l-UAE/Time/Temp.)	TPC (mg/g DM) *			
	212 µm		<1000 µm	
	Silent	US	Silent	US
C-horn/45 min/45 °C	27.09 ^a^	34.00 ^a^	24.5 ^a^	31.89 ^a^
I-horn/30 min/RT	26.11 ^a^	34.08 ^a^	19.06 ^b^	26.03 ^b^
I-horn/30 min/45 °C	26.81 ^a^	29.11 ^b^	22.17 ^a^	28.24 ^b^

^a,b^ Tukey’s post-hoc test groups (each comparison has been performed within every column independently; Bonferroni adjustment for three tests and three estimates). * TPC are expressed as average values of GAE on DM (SE 0.81, confidence level 0.95).

## Appendix A: Model, Statistics and HPLC

**Table A1:** Linearization, I-horn.

Extr. Time (min)	t/TPC	
	RT	45 °C
5	0.4604	0.3185
8	0.5626	0.4670
10	0.6549	0.5858
12	0.7614	0.5326
15	0.8762	0.5699
30	1.1525	1.1223
45	1.6924	1.5146

**Table A2:** Linearization, C-horn.

Extr. Time (min)	t/TPC
5	0.3119
8	0.3685
10	0.4357
12	0.4914
15	0.5714
30	1.0623
45	1.4111

**Table A3:** Quantification of polyphenols (mg/g on dried extract) by HPLC-DAD analyses in l-UAE (H_2_O, cup-horn, 45 min, 45 °C) and conventional hydroalcoholic extracts (EtOH/H_2_O 60:40, 23 min, 95 °C, reflux).

Method		Compound	RT (min)	λ max (nm)	λ quant. (nm)	l-UAE (mg/g DE)	Conv. (mg/g DE)
Pol	ST	Gallic acid	10.7	272	280	5.70	3.86
	UV	Quercetin deriv. (3-glucuronide?)	28.12	257, 355.4	340	0.905 ^a^	7.34 ^a^
	UV	Quercetin deriv. (rutin?)	30.1	265, 350.6	340	1.09 ^a^	-
	ST	ε-viniferin	35.07	233.5, 324.4	340	-	2.89
Cat	ST	Catechin	16.2	234.7, 280.6	280	3.98	5.58

^a^ Expressed as quercetin equivalents, mg/g DE.

**Table A4:** Identification of polyphenols by HPLC-DAD analyses in l-UAE (H_2_O, cup-horn, 45 min, 45 °C) and conventional hydroalcoholic extracts (EtOH/H_2_O 60:40, 23 min, 95 °C, reflux).

Method		Compound	RT (min)	λ max (nm)	l-UAE	Conv.
Pol	UV	Cinnamic acid deriv.	22.8	295, 329	✓	-
	UV	Catechin deriv. (OPC?)	23.4	235.9, 280.6	✓	-
	UV	Cinnamic acid deriv.	23.57	295, 329	-	✓
	UV	Catechin deriv. (OPC?)	24.02	234.7, 285.3	✓	✓
	UV	Catechin deriv. (OPC?)	24.38	235.9, 281.8	✓	✓
	UV	Catechin deriv. (OPC?)	24.77	234.7, 280.6	✓	✓
	UV	Cinnamic acid deriv.	25.16	295, 314.9	✓	✓
	UV	Catechin deriv. (OPC?)	26.85	235.9, 280.6	✓	-
	UV	Unknown flavonoid	27.5	258.2, 359.6	✓	✓
	UV	Isorhamnetin	28.9	257, 356.5	✓	✓
	UV	Unknown flavonoid	29.6	265, 349.4	✓	-
	ST	Veratric acid	30.5	261.8, 299.5	✓ ^a^	-
	UV	Kaempherol deriv.	34.2	255.9, 368.2	-	✓
Cat	UV	Catechin deriv. (OPC?)	11.9	233.5, 280.6	✓	- ^a^
	UV	Catechin deriv. (OPC?)	13.8	232.4, 274.7	✓	✓
	UV	Catechin deriv. (OPC?)	15.7	234.7, 280.6	✓	-
	UV	Catechin deriv. (OPC?)	20.6	234.7, 280.6	-	✓
	UV	Catechin deriv. (OPC?)	23.0	234.7, 283	✓	✓

✓: identified; -: not detected; ^a^ Overlapping of broad peaks as a result of OPC derivatives.

**Table A5:** Retention times (RTs), λ max, λ for eq., equation curves, linearity ranges, R^2^, limit of detection (LOD), and limit of quantification (LOQ) for all of the identified standard compounds (ST).

Compound	RT (min)	λ max (nm)	λ for eq. (nm)/HPLC method	Equation Curve (mg/mL)	Lin. Range (mg/mL)	*R* ^2^	LOD (mg/mL)	LOQ (mg/mL)
Gallic acid	10.7	272	280 (Pol)	y = 57569151.7x	0.0012–0.582	0.9997	0.0005	0.0012
Quercetin ^a^	- ^a^	355	340 (Pol)	y = 62735637.1x – 8816.1	0.0012–0.100	1.0000	0.0006	0.0012
(+)-Catechin	16.2	233.5, 281.6	280 (Cat)	y = 16107243.3x – 131.6	0.002–0.100	0.9993	0.001	0.002
ε-viniferin	35.1	233.5, 325.6	340 (Pol)	y = 77687802.0x – 155,501	0.0011–0.043	0.9998	0.0005	0.0011

^a^ Quercetin was used as the reference compound for the quantification of its derivatives, the R_t_ is therefore different from those of the compounds in the extracts.

**Table A6:** Linearization, S-UAE.

Extr. Time (min)	*t*/TPC
2	0.5862
5	0.6338
8	0.7064
15	0.8744
30	1.1758
60	1.9428
90	2.7511
120	3.6806

**Table A7:** ANOVA table on the TPC response (mg/g DM) reported in Table 5.

ANOVA Ways	Df	Sum Sq	Mean Sq	*F* Value	Pr(>F)	^a^
Time	1	438.98	438.98	310.9257	5.887·10^−11^	***
Granulometry	1	312.25	312.25	221.1629	5.703·10^−10^	***
Time:Granulometry	1	0.09	0.09	0.0646	8.03·10^−1^	-
Residuals	14	19.77	1.41			

^a^ Signif. codes: 0 ‘***’ 0.001 ‘**’ 0.01 ‘*’ 0.05 ‘.’ 0.1 ‘-’ 1.

**Table A8:** ANOVA table on the TPC response (mg/g DM) reported in Table 4 and Table 6.

ANOVA Ways	Df	Sum Sq	Mean Sq	F Value	Pr(>F)	^a^
Extr. cond.	2	68.57	34.28	17.43	2.109·10^−^^5^	***
US	1	353.63	353.63	179.82	1.213·10^−^^12^	***
Granulometry	1	160.15	160.15	81.44	3.509·10^−^^9^	***
Extr. cond.: US	2	19.68	9.84	5.005	1.525·10^−^^2^	*
Extr. cond.: Granulometry	2	50.20	25.10	12.7627	1.68·10^−^^4^	***
US: Granulometry	1	2.64	2.64	1.343	2.579·10^−^^1^	-
Extr. cond.: US: Granulometry	2	8.94	4.47	2.274	1.247·10^−^^1^	-
Residuals	24	47.20	1.97			

^a^ Signif. codes: 0 ‘***’ 0.001 ‘**’ 0.01 ‘*’ 0.05 ‘.’ 0.1 ‘-’ 1.
